# Nonantibiotic Effects of Fluoroquinolones in Mammalian Cells*

**DOI:** 10.1074/jbc.M115.671222

**Published:** 2015-07-23

**Authors:** Sujan Badal, Yeng F. Her, L. James Maher

**Affiliations:** From the Department of Biochemistry and Molecular Biology, Mayo Clinic, Rochester, Minnesota 55905

**Keywords:** antibiotic action, collagen, dioxygenase, epigenetics, iron, fluoroquinolone

## Abstract

Fluoroquinolones (FQ) are powerful broad-spectrum antibiotics whose side effects include renal damage and, strangely, tendinopathies. The pathological mechanisms underlying these toxicities are poorly understood. Here, we show that the FQ drugs norfloxacin, ciprofloxacin, and enrofloxacin are powerful iron chelators comparable with deferoxamine, a clinically useful iron-chelating agent. We show that iron chelation by FQ leads to epigenetic effects through inhibition of α-ketoglutarate-dependent dioxygenases that require iron as a co-factor. Three dioxygenases were examined in HEK293 cells treated with FQ. At sub-millimolar concentrations, these antibiotics inhibited jumonji domain histone demethylases, TET DNA demethylases, and collagen prolyl 4-hydroxylases, leading to accumulation of methylated histones and DNA and inhibition of proline hydroxylation in collagen, respectively. These effects may explain FQ-induced nephrotoxicity and tendinopathy. By the same reasoning, dioxygenase inhibition by FQ was predicted to stabilize transcription factor HIF-1α by inhibition of the oxygen-dependent hypoxia-inducible transcription factor prolyl hydroxylation. In dramatic contrast to this prediction, HIF-1α protein was eliminated by FQ treatment. We explored possible mechanisms for this unexpected effect and show that FQ inhibit HIF-1α mRNA translation. Thus, FQ antibiotics induce global epigenetic changes, inhibit collagen maturation, and block HIF-1α accumulation. We suggest that these mechanisms explain the classic renal toxicities and peculiar tendinopathies associated with FQ antibiotics.

## Teaser

**Background:** Fe(II)-dependent dioxygenases regulate epigenetic control, collagen maturation, and HIF degradation.

**Results:** Iron chelation by fluoroquinolone antibiotics results in DNA and histone hypermethylation, suppression of collagen prolyl hydroxylation, and inhibition of HIF mRNA translation.

**Conclusion:** Dioxygenase inhibition may explain renal toxicity and tendinopathy side effects of fluoroquinolones.

**Significance:** This study suggests mechanisms for obscure fluoroquinolone-associated side effects and possible novel applications of these antibiotics as HIF antagonists.

## Introduction

**FIGURE 1.**
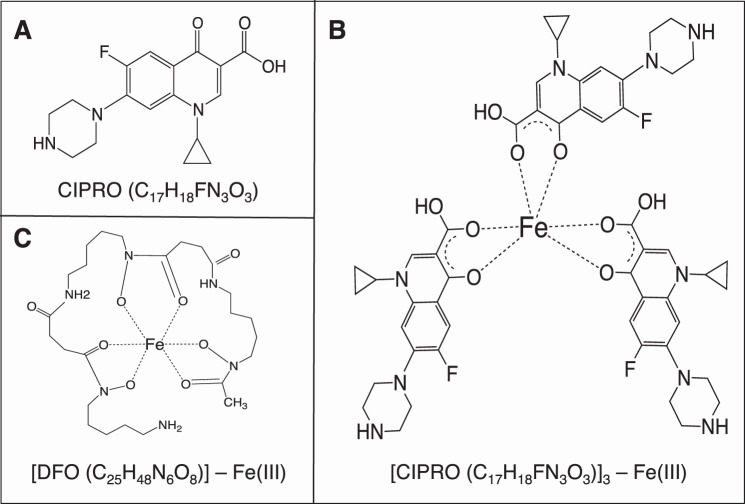
**Structures of compounds and complexes under study.** *A,* ciprofloxacin. *B,* ternary chelate of CIPRO and Fe(III). *C,* deferoxamine chelate with Fe(III).

Food and Drug Administration-approved antimicrobial drugs are designed to target pathogenic microorganisms with minimal effects on the host. However, nonantibiotic effects of antimicrobial agents are well known (1), due to unexpected interactions with cellular pathways. Generalized adverse effects (2–4) are common to most antimicrobials, balancing against benefits (5–11). Here, we investigate the interaction of relevant concentrations of fluoroquinolone (FQ)^3^ antibiotics ciprofloxacin (CIPRO; Fig. 1*A*), norfloxacin (NOR), and enrofloxacin (ENRO) with a cultured human embryonic kidney cell line, revealing previously unreported enzyme inhibition effects that may explain toxicities associated with FQ treatment.

FQs are popular synthetic broad-spectrum antibiotics that exert their antimicrobial effect by preventing energy-dependent negative supercoiling of bacterial DNA through gyrase inhibition (12). FQs are effective agents that target both Gram-negative and Gram-positive bacteria and are recommended for severe bacterial infections, including multidrug-resistant infections (13). FQ side effects have been widely studied (14–19). However, the molecular mechanisms underlying these toxicities remain to be elucidated. One such peculiar FQ side effect is tendinopathy (15, 20). The majority (>85%) of FQ-associated tendinopathies occur within a month of initial FQ therapy, with a 3-fold higher chance of tendon rupture within the first 90 days of exposure (21). In rare cases of patients with pre-existing musculoskeletal disorders, FQ therapy has been linked to tendinopathy as early as a few hours after administration to as late as 6 months after discontinuing medication (22). Although compromised collagen integrity after FQ treatment is well recognized in animal models (17, 22, 23), the underlying mechanism is unknown. Some studies report association of enhanced matrix metalloprotease (23, 24) or collagenase (25) expression associated with FQ-induced tendinopathy. However, a direct link to defects in collagen, a protein that accounts for greater than 6% of muscle mass (26), is still obscure.

FQ-associated nephrotoxicity is also well documented (27–35). Past clinical studies on patients receiving FQ therapy have revealed a strong association with acute renal failure involving interstitial nephritis (27, 32, 34), acute tubular necrosis (29), and more recently crystalluria (33, 35). These complications are often attributed to immune-mediated allergic hypersensitivity to FQ antibiotics, with reversal after discontinuation of drug treatment (31, 35). Although considerable clinical evidence for FQ-associated nephropathy is available, detailed cellular effects of these antibiotics leading to nephritis are not well understood. Appreciating the mechanism of pathological side effects is important for improving our understanding of FQ-associated nephrotoxicity and for illuminating potential complications. Here, we provide evidence for new mechanisms of FQ toxicity involving renal cell epigenetics, impaired collagen maturation, and suppression of the hypoxia-inducible factor, HIF-1α. We show that at least some of these effects are due to the powerful iron-chelating property of FQ drugs.

An intrinsic FQ characteristic is the propensity to bind to metal cations (36–38). This is due to the electronegative oxygen atoms in the adjacent pyridone and carboxylate moieties (Fig. 1) of all quinolone derivatives (39). The potential for metal chelation by FQ suggests multiple toxic effects on cells. Here, we focus on FQ effects on a class of Fe(II)-dependent enzymes known as 2-ketoglutarate (2-KG)-dependent dioxygenases (40). The first and best characterized 2-KG dioxygenase is prolyl 4-hydroxylase, which catalyzes the post-translational hydroxylation of proline residues in collagen (41, 42). Other Fe(II)-dependent dioxygenases include HIF-1α-prolyl hydroxylase dioxygenase (PHD), jumonji domain histone demethylases (JMHD), and TET methylcytosine dioxygenase 1 (TET1), responsible for hydroxylation of the HIF-1α transcription factor, histone demethylation, and DNA demethylation, respectively. Here, we test the hypothesis that all of these dioxygenases are subject to inhibition by the iron-chelating properties of FQ antibiotics.

In contrast to these dramatic epigenetic changes consistent with the predicted effects of iron chelation on dioxygenases, we report an unpredicted result in the case of HIF-1α. Here, dioxygenase inhibition should stabilize HIF-1α by protecting it from prolyl hydroxylation (43). In fact, FQ treatment has the *opposite* effect, strongly suppressing HIF-1α accumulation.

Thus, we suggest that iron chelation by FQ antibiotics inhibits α-KG-dependent collagen prolyl 4-hydroxylase and other dioxygenase enzymes, perhaps explaining FQ side effects, including spontaneous tendon ruptures (44). In addition, FQ-induced epigenetic modifications uncovered here may explain aspects of FQ nephrotoxicity. Finally, our unexpected observation of FQ-induced HIF-1α loss suggests the possible use of FQ drugs in cancer therapy (45–48).

## Experimental Procedures

### 

#### 

##### Cell Culture

Human embryonic kidney (HEK293) cells were cultured under physiologically relevant oxygen conditions (49) as follows: 37 °C, 90% humidity, 5% CO_2_, 2% oxygen balanced by N_2_ in DMEM (Gibco) containing 10% FBS and 1% penicillin/streptomycin.

##### Iron Competition Assay

The universal siderophore assay of Schwyn and Neilands (50) was used to measure the iron chelating activity of FQ antibiotics. Deferoxamine mesylate (DFO; Calbiochem), a siderophore produced by *Streptomyces pilosus*, was used as the positive control. Chrome azurol S (CAS) assay solution (100 ml) was prepared with the following final concentrations: CAS (Sigma, 199532; 0.15 mm), hexadecyltrimethylammonium bromide (Sigma, 1102974; 0.6 mm), iron (III) chloride hexahydrate (Sigma, 236489; 0.015 mm from a stock dissolved in 10 mm HCl), and 4.3 g of anhydrous piperazine (Sigma, P45907) dissolved in 6.25 ml of 12 m HCl and pH adjusted to 5.6. The solution was stored in the dark at 4 °C. Iron-binding reactions were conducted in triplicate with 0.5-ml aliquots of the CAS assay solution and various concentrations of antibiotics to a total volume of 700 μl. Test samples were incubated at room temperature, with slow rotation for 30 min, and then transferred to a 96-well plate for absorbance measurement (630 nm) using a microplate spectrophotometer. The apparent half-maximal inhibitory concentration (IC_50_) for iron complexes was estimated.

Stoichiometry determination was conducted based on Schwyn and Neilands (50) assay as described above. In a typical assay, the concentration of iron (Fe^3+^) was held constant, and increasing concentrations of test compounds required to quench the absorbance of CAS-iron complex was added. Because tested drug concentrations are all well above the equilibrium dissociation constant for complex formation, the [compound]/[Fe^3+^] ratio required for complete quenching in such titrations gives the compound/Fe^3+^ binding stoichiometry.

##### Cell Culture Treatment with FQ, DFO, CoCl_2_, and Ferric Citrate

10 mm stock solutions of CIPRO, ENRO, and NOR (Sigma, 17850, 17849, and N9890, respectively) were prepared by dissolving FQs in 0.01 n HCl. HEK293 cells were cultured in 10-cm dishes to 70% confluence prior to treatment with FQs at the indicated final concentrations for the indicated times. In some experiments, cells were treated with either 100 μm DFO (Sigma, D9533) or CoCl_2_ for 4 h. An equal volume of 0.01 n HCl (NT) was added to a separate dish of cells as negative control. In co-treatment experiments, cells were first treated with CIPRO for 30 min followed by addition of the indicated concentrations of ferric citrate (Sigma, F3388) for 4 h. Cells viability was determined using trypan blue dye exclusion.

##### Immunoblotting

Western blot analyses were performed by growing cells in 10-cm dishes, harvesting, and lysis with RIPA buffer containing 1× protease inhibitor mixture (Roche Applied Science) and 1× phosphatase inhibitor (Thermo Scientific). Cell lysate was agitated on ice for 20 min prior to centrifugation at 14,000 rpm for 15 min at 4 °C in a microcentrifuge. Extracts were analyzed by electrophoresis through 10% BisTris-polyacrylamide gels under reducing conditions with detection by Western blotting using anti-HIF-1α antibody (BD Biosciences 610958, 1:1000), anti-HIF-2α antibody (Novus Biologicals 100-122, 1:1000), anti-H3K9me2 antibody (Abcam 1220, 1:1000), anti-H3K9me3 antibody (Millipore 07-442, 1:500), anti-H3K27me2 antibody (Abcam 24684, 1:1000), anti-H3K27me3 antibody (Millipore 05-1951, 1:15,000), anti-H3 antibody (Santa Cruz Biotechnology 10809, 1:1000), anti-HDAC6 antibody (Cell Signaling 7558S, 1:1000), anti-JMJD2D antibody (Abcam 93694, 1:200), anti-TET1 antibody (Abcam 156993, 1:1000), or anti-actin antibody (Sigma A2066, 1:500). Secondary antibodies were anti-rabbit- and anti-mouse-conjugated to a horseradish peroxidase (Promega, 1:15,000), and signals were developed using an ECL plus kit (Pierce).

##### Genomic DNA Extraction and Hydrolysis

A Qiagen genomic DNA extraction kit was used to harvest genomic DNA from cells. The manufacturer's instructions were followed with minor changes as described (51). Briefly, the cell pellet was lysed with C1 buffer and subjected to centrifugation at 1000 rpm for 10 min at 4 °C in a clinical centrifuge. Pelleted nuclei were resuspended in C1 buffer with centrifugation for 5 min at 4 °C. G2 buffer was used to lyse the nuclear membranes. RNase A solution (Thermo Scientific, final concentration 10 mg/ml) and proteinase K solution (Sigma, final concentration 10 mg/ml) were added to the lysed nuclei and incubated overnight at 55 °C. Subsequent purification steps were according to the manufacturer's instructions. Genomic DNA was washed with 70% ethanol, resuspended in water, and stored at −20 °C. Three micrograms of genomic DNA were hydrolyzed to mononucleosides as described (52). The resulting 40-μl mixture contained 3 μg of DNA, 1× micrococcal nuclease buffer (New England Biolabs), 400 mm MgCl_2_, 4 mm ZnCl_2_, 20 units of deoxyribonuclease I (New England Biolabs), 2000 units of micrococcal nuclease I (New England Biolabs), 5 units of antarctic phosphatase (New England Biolabs), and 0.4 units of snake venom phosphodiesterase. Reactions were incubated overnight at 37 °C.

##### LC-MS Analysis of Nucleosides

LC-MS was performed by loading 0.6 μg of mononucleosides from digested genomic DNA onto a C18 analytical reverse phase column (Phenomenex-C18 1.0 × 250 mm) using an Agilent series 1100 instrument (Agilent Technologies) with mobile phase A (0.05 m ammonium formate (pH 5.4); Sigma, 17843) and mobile phase B (methanol) at a flow rate of 0.05 ml/min and absorbance at 277 nm. The following gradient program was used: 0 min, 2% B; 18 min, 10% B; 30 min, 25% B; 35 min, 2% B, and 60 min, 2% B. Mass spectrometry was performed as described previously (52). Briefly, the HPLC effluent was connected in-line to a mass spectrometer (MSD-TOF, Agilent Technologies) operated in positive ion mode. The MS conditions were as follows: nebulizer 20 p.s.i., dying gas 7 liters/min, gas temperature 325 °C; fragmentor 45 V, Oct 1 DC 37.5 V; Oct RF 250 V. All data were analyzed using Agilent MassHunter quantitative analysis software.

##### JNK1 Activity Assay

A JNK activity screening kit (Abcam, ab65784) was used to determine JNK1 enzyme activity in an *in vitro* kinase reaction. Briefly, JNK was captured from total cell lysate using an N-terminal c-Jun(1–79) fusion protein bound to glutathione-Sepharose beads. After removal of nonspecifically bound proteins by several washes, the JNK1/c-Jun beads were incubated in the kinase assay buffer for 30 min at 30 °C in the presence of various concentrations of CIPRO. The kinase reaction was then initiated by addition of ATP. c-Jun phosphorylation was measured by Western blot analysis using an anti-phospho-c-Jun antibody.

##### HSP90 Immunoprecipitation

Total lysate (0.4 mg) from control and drug-treated cells was incubated overnight with 5 μl of anti-HSP90 antibody (Abcam, 13495) at 4 °C. Protein A/G magnetic beads (20 μl) were added to the mixture with incubation (gentle rotation) for 4 h at 4 °C. Immunoprecipitated samples were washed and subjected to Western blot analysis with anti-pan-acetyl-lysine antibody (Cell Signaling, 94415) and anti-HSP90 antibody.

##### Quantitative Real Time PCR

HIF-1α, HSP90, HDAC6, P4HA1, and LH1mRNA levels were quantified by qRT-PCR analysis. HEK293 cells treated with CIPRO or DFO in 2% oxygen for 24–48 h were harvested, and total RNA was extracted using an RNeasy micro kit (Qiagen, 74004). cDNA was synthesized from total RNA with oligo(dT) primers using SuperScript III first strand synthesis system (Invitrogen, 18080-051). Primer efficiency was determined by validating performance with a standard curve (*C_t_* value *versus* log DNA dilution) with a correlation coefficient (*R*^2^) of 1 corresponding to 100% primer efficiency. For sample mRNA quantification, 20 ng of cDNA template was prepared with 1 μm forward and reverse primers and a master mix from the LightCycler TaqMan master kit (Roche Applied Science, 04535286001). β-Actin was used as an internal control, and results were analyzed relative to Δ*C_t_* values. All computations were done using the Pfaffl method for relative RT-PCR analysis (53). Primer sequences were as follows: forward 5′-CAGAGCAGGAAAAGGAGTCA and reverse 5′-AGTAGCTGCATGATCGTCTG for HIF-1α; forward 5′-GCTACTGCCATCCAATCGAG and reverse 5′-CTCTCCTATGTGCTGGCCTT for VEGF; forward 5′-GGTGTCTGATGATGAAGACGAG and reverse 5′-CACCTCCAGCTCCTTCAGTT for HSP90; forward 5′-CCCAATCTAGCGGAGGTAAA and reverse 5′-CCTCACCTGTCATCCCAGAG for HDAC6; forward 5′-GTGGATTACCTGCCAGAGAGACA and reverse 5′-CTCGGCTCAGCCTTGGTTT for P4HA1; forward 5′-GGAACCTGGCCTATGACACCCT and reverse 5′-TGCCATGCTGTGCCAGGAACT for LH1; forward 5′-CAAATATGTACGGGGCAACC and reverse 5′-TACTCAGACCTGGGGGTACG for JMJD2D; and forward 5′-GCTCTCATGGGTGTCCAATTGCT and reverse 5′-ATGAGCACCACCATCACAGCAG for TET1.

##### Metabolic Labeling

2 × 10^6^ cells were seeded into 10-cm dishes and cultured for 24 h at 2% O_2_, washed, and then incubated with methionine-free DMEM (Gibco) containing 10% dialyzed serum for 18 h. Cells were then incubated with the indicated concentrations of DFO or CIPRO for 30 min. Newly synthesized proteins were radiolabeled by addition of 5 μCi of [^35^S]methionine (l-[^35^S]methionine; PerkinElmer Life Sciences, 0.3 mCi/ml). After the cells were pulsed for 40 min, cells were washed twice with PBS before the addition of DMEM containing l-methionine. Cells were harvested in cell lysis buffer (50 mm HEPES-KOH (pH 8.0), 2 mm EDTA (pH 8.0), 150 mm NaCl, 1% Triton X-100, 0.1% sodium deoxycholate, 0.2% SDS, 1 mm PMSF, and mixture protease inhibitor tablet) on ice. Total lysate (500 μg) was incubated with 5 μl of anti-HIF1α monoclonal antibody (BD Biosciences) for 4 h at 4 °C. Protein A/G magnetic beads (20 μl) were added with incubation and gentle rotation for 4 h at 4 °C. Immunoprecipitated samples were resuspended in sample buffer and heated at 95 °C for 5 min. Radiolabeled HIF-1α protein was assessed using SDS-PAGE analysis and storage phosphorimaging (Typhoon system).

##### Collagen Hydroxyproline Quantification

Total hydroxylated proline residues in collagen were determined using the hydroxyproline assay kit (Chrondrex, 6017) as directed by the manufacturer. Briefly, HEK293 cells were stimulated with 50 μg/ml ascorbate to stimulate collagen production (54, 55) and treated at 0, 24, and 48 h with 100, 150, and 250 μm FQ or DFO. Cells were washed three times with PBS to remove any residual drug prior to each treatment. At 72 h, HEK293 cells were harvested, counted, and lysed with 100 μl of 5 n HCl in polystyrene tubes (Falcon, 352058). The cell lysate was incubated in 60 °C for 24 h prior to centrifugation at 10,000 rpm for 3 min in a microcentrifuge. The supernatant (lysate) was analyzed to calculate hydroxyproline levels by colorimetric analysis using a standard curve.

## Results

### 

#### 

##### FQs Are Potent Iron Chelators

**FIGURE 2.**
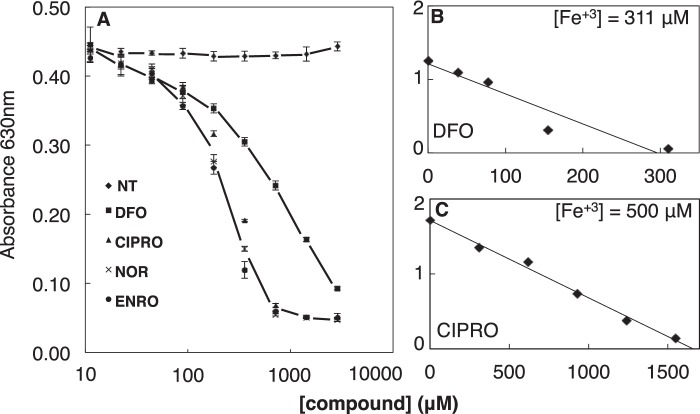
**FQs are potent iron chelators.** *A,* iron chelation as determined by CAS competition assay. Iron binding stoichiometries were determined for DFO (positive control) (*B*) and CIPRO with iron at indicated concentrations (higher than *K_d_*) (*C*). Data are representative of *n* ≥ 3 independent experiments. *NT*, not treated (diluent only).

Metal binding by FQ has been described previously (38). Physiochemical and spectroscopic analysis suggest a 3:1 (CIPRO/Fe^3+^) coordination complex involving pyridone and carboxylate oxygen atoms (Fig. 1) present in all quinolones (39). We verified and quantitated iron binding by three FQ antibiotics (CIPRO, NOR, and ENRO). The colorimetric siderophore detection assay of Schwyn and Neilands (50) was employed. FQ competition with CAS for iron binding reduces the absorbance of the assay solution at 630 nm. Strong iron chelation was observed for the three tested FQ drugs as well as the DFO-positive control (Fig. 2).

To confirm the stoichiometry of the FQ-iron complex, we utilized the Schwyn and Neilands (50) assay to determine the concentration of FQs necessary to quench the absorbance of a known concentration of the CAS-iron complex. Analysis was performed for CIPRO and DFO (positive control known to form a 1:1 complex with iron). The results (Fig. 2, *B* and *C*) confirm that DFO and CIPRO form 1:1 and 3:1 complexes, respectively (Fig. 1, *B* and *C*). IC_50_ values were then estimated (Table 1). The three tested FQ drugs chelate iron at least as well as DFO, a well known siderophore in clinical use. This verification of strong iron chelation by FQ antibiotics led us to explore the biological effects of FQ on iron-dependent enzymes in cultured cells.

**TABLE 1:** **Iron binding by ligands**

Ligand	Stoichiometry (ligands/iron)	Mean IC_50_*^a^*
		μ*m*
Chrome azurol S	2	
Ciprofloxacin	3	52 ± 20
Norfloxacin	3	44 ± 15
Enrofloxacin	3	41 ± 20
Deferoxamine	1	360 ± 25

*^a^* Mean IC_50_ values were from *n* ≥ 3 iron binding assays.

##### FQs Inhibit Jumonji Domain Histone Demethylase and TET Demethylase Activities in Cultured Cells

**FIGURE 3.**
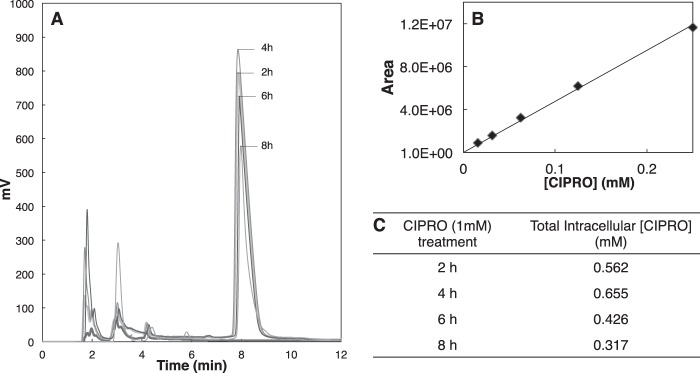
**FQ cellular uptake.** *A,* HPLC of FQ uptake into HEK293 cells as a function of time. *B,* standard curve for CIPRO quantitation. *C,* calculated intracellular CIPRO concentrations as a function of time.

All 2-KG-dependent dioxygenases initially hydroxylate substrates using chemistry that requires Fe^2+^, 2-KG, and O_2_ (56, 57) in ordered tri-tri reactions producing succinate and CO_2_ as by-products (40, 41). Decreases in Fe^2+^ (58), 2-KG (59), or O_2_ (60, 61) inhibit the activities of these enzymes. To assess whether iron chelation by FQ inhibits 2-KG-dependent dioxygenases in cultured kidney cells (a model of kidney exposure to FQ after antibiotic treatment), we treated HEK293 cells with CIPRO, NOR, or ENRO for 2, 4, or 6 h in hypoxia (2% O_2_). High FQ concentrations (>1 mm) were toxic after 8 h. In contrast, cells tolerated FQ treatment for 4 h. This time point was chosen because HPLC analysis showed maximal drug uptake into cells (Fig. 3) without cell detachment and with greater than 95% cell viability as detected by trypan blue dye exclusion.

**FIGURE 4.**
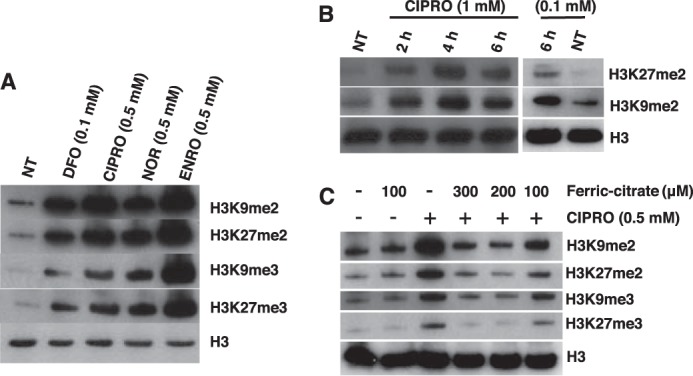
**Accumulation of histone methylation in HEK293 cells treated with the indicated FQ.** *A,* H3K9me2, H3K27me2, H3K9me3, and H3K27me3 abundance by Western blotting after 4 h of treatment with the indicated FQs (0.5 mm). *B,* H3K27me2 and H3K9me2 abundance after 2, 4, and 6 h of treatment with CIPRO (0.1 or 1 mm). *C,* FQ inhibition of jumonji domain histone demethylases in cell culture is rescued by inclusion of the indicated concentrations of ferric citrate (C_6_H_5_FeO_7_). NT, diluent only.

**FIGURE 5.**
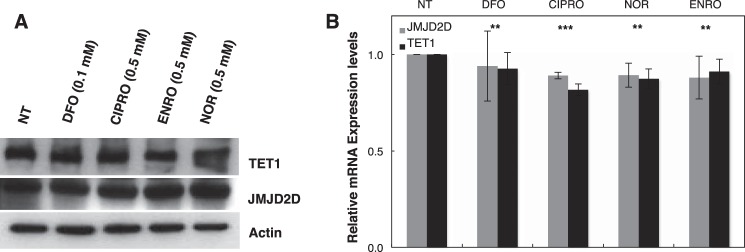
**JMJD2D and TET1 enzyme levels in HEK293 cells treated with indicated FQ.** *A,* TET1 and JMJD2D abundance by Western blotting after 4 h of treatment with the indicated FQs (0.5 mm). *B,* TET1 and JMJD2D mRNA expression levels in cells after 24 h of indicated FQ treatment. Data are means ± S.D. reflecting *n* ≥3 independent experiments. Statistical analysis by paired *t* test (compared with NT control, diluent only) is shown. Statistical significance (**, *p* ≤ 0.05; ***, *p* ≤ 0.005) is shown.

**FIGURE 6.**
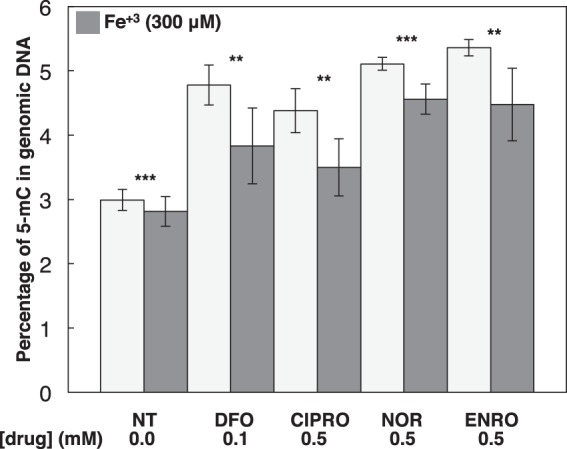
**FQ inhibition of the TET Fe/O_2_/2-KG-dependent dioxygenase enzyme is rescued after ferric citrate treatment.** HEK293 cells were treated with proposed drugs or co-treated with 300 μm ferric citrate. Data are means ± S.D. reflecting *n* ≥3 independent experiments. Statistical analysis by paired *t* test (compared NT control, diluent only) is shown. Statistical significance (**, *p* ≤ 0.05; ***, *p* ≤ 0.005) is shown.

We evaluated histone and DNA demethylation by the JMHD and TET1 families of dioxygenases, respectively. These dioxygenases play roles in determining the epigenetic status of chromatin. JMHD catalyzes the removal of methyl groups from histone tails. Its inhibition leads to increased global histone methylation. TET1 catalyzes the first step of cytosine demethylation. Inhibition of TET1 results in global accumulation of 5-methylcytosine in genomic DNA. Western blot analysis showed accumulation of H3K9me2, H3K9me3, H3K27me2, and H3K27me3 in cells treated with (0.1–1 mm) CIPRO, NOR, and ENRO compared with control (Fig. 4). Furthermore, analysis of protein and mRNA expression for histone demethylase (JMD2D) and TET1 showed little or no effect of FQ treatment (Fig. 5), confirming that loss of these activities is due to enzyme inhibition. LC-MS analysis also showed an increase in 5-methylcytosine levels in the genomic DNA of cells treated with 0.5 mm CIPRO, NOR, or ENRO for 4 h in hypoxia compared with controls (Fig. 6). Importantly, these inhibitory effects were reversible by co-treatment of cells with ferric citrate, pointing specifically to iron chelation as the mechanism of dioxygenase inhibition (Fig. 4*B*, 6).

##### FQs Inhibit COL-P4H Enzyme Activity and Suppress COL-P4H and lysyl hydroxylase mRNA Levels

**FIGURE 7.**
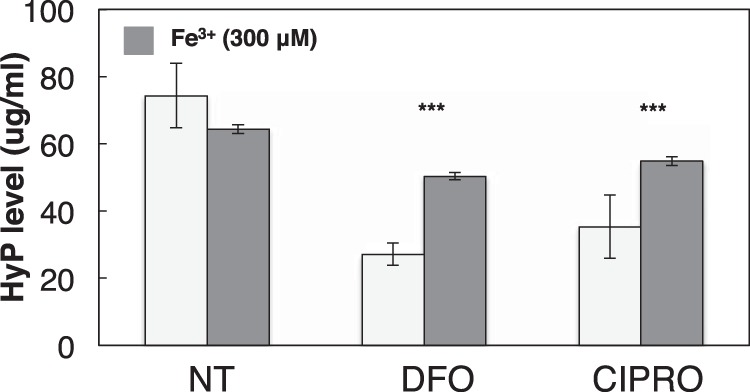
**FQ treatment inhibits collagen proline hydroxylation.** HEK293 cells were co-treated with 50 μg/ml ascorbate and either NT, DFO, or CIPRO for 72 h as follows: 0 h, 100 μm; 24 h, 150 μm; and 48 h, 250 μm. At 72 h, cells were harvested and processed for quantification of hydroxyproline (*HyP*) in total collagen. Similarly, 300 μm ferric citrate was added to NT, DFO, or CIPRO cell cultures, and hydroxyproline levels were assessed accordingly. Data are normalized to cell number and represent at least three independent experiments. Data are reported as means ± S.D. Statistical analysis was performed using paired *t* test (compared with NT). Significant difference (***, *p* ≤ 0.005) is shown. NT (diluent only).

Collagen maturation involves extensive prolyl hydroxylation catalyzed by iron-dependent dioxygenases (62). These collagen prolyl 4-hydroxylases are located within the lumen of the endoplasmic reticulum and catalyze the formation of 4-hydroxyproline by the hydroxylation of prolines in -Xaa-Pro-Gly- sequences in collagens and more than 15 other proteins that have collagen-like domains (62–64). Thus, prolyl 4-hydroxylases have a central role in the maturation of collagens, as 4-hydroxyproline residues are essential for the formation of the collagen triple helix. To assess FQ effects on collagen maturation, HEK293 cells were treated for 72 h with increasing FQ concentrations. FQ-treated cells showed decreased collagen prolyl hydroxylation relative to untreated cells. Similarly, treatment with DFO shows comparable reduction of hydroxylated proline residue as expected. Additionally, co-treatment of cells with ferric citrate (300 μm) caused partial reversal of effects seen during FQ treatment, reinforcing the role of iron chelation in dioxygenase inhibition (Fig. 7).

**FIGURE 8.**
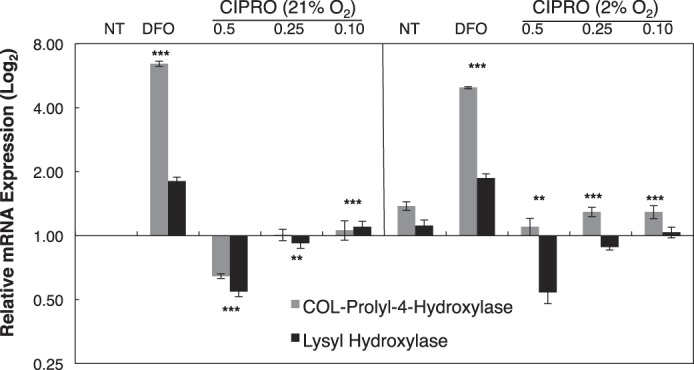
**FQ represses transcription of genes encoding enzymes involved in collagen synthesis and maturation.** Collagen prolyl 4-hydroxylase and lysyl hydroxylase mRNA expression levels in cells treated with CIPRO at 21 or 2% O_2_ after 24 h of treatment. Data are means ± S.D. representative of *n* ≥ 3 independent experiments. Statistical significance (**, *p* ≤ 0.05; ***, *p* ≤ 0.005). NT (diluent only).

Surprisingly, FQ treatment also led to decreased COL-P4H1 and LH1 mRNA levels (Fig. 8). Similar to P4H, lysyl hydroxylases support collagen maturation by hydroxylating lysine residues that serve as attachment sites for galactose and glucosylgalactose and as precursors of the cross-linking process that gives collagen its tensile strength (65). As discussed below, FQ down-regulation of COL-P4H1 and LH1 mRNA levels may reflect the role of HIF-1α in driving expression of these genes (66).

These results suggest, for the first time, that FQ treatment can cause unanticipated epigenetic effects. Moreover, we suggest that the well established linkage between FQ treatment and tendinopathy reflects impairment of collagen maturation by FQ. We suggest that it is the inhibition of collagen prolyl 4-hydroxylases by FQ-mediated iron chelation and the repression of collagen P4H1 and LH1transcription that underlie the peculiar tendinopathy side effects of FQ antibiotics.

##### Unexpected Suppression of HIF-1α after FQ Treatment

**FIGURE 9.**
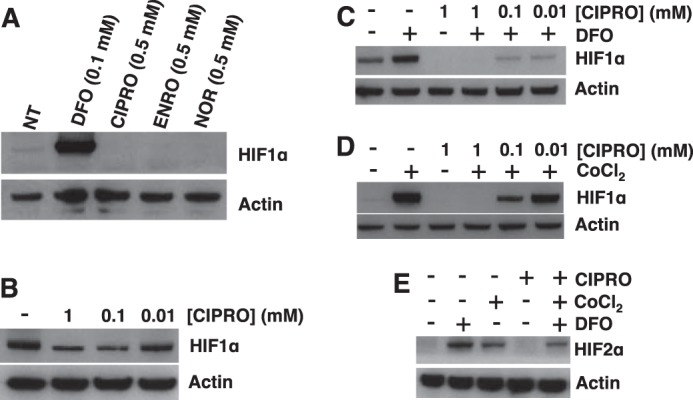
**FQs suppress HIF-1α in HEK293 cells.** *A,* relative HIF-1α levels assessed by Western blotting in HEK293 cells with 0.5 mm FQ treatment for 4 h in hypoxia (2% oxygen). *B,* HIF-1α levels in HEK293 cells treated with CIPRO in hypoxia. HIF-1α status in HEK293 cells with co-treatment of CIPRO and DFO (*C*) or CoCl_2_ for 4 h in hypoxia (*D*) is shown. *E,* HIF-2α levels in HEK293 cells with co-treatment of CIPRO and DEF or CoCl_2_ in hypoxia for 4 h. 100 μm DFO (positive control) or CoCl_2_ (positive control) was used in the co-treatment experiments. NT (diluent only).

PHD is a 2-KG-dependent dioxygenase that determines the fate of HIFs in cells. In normoxia, PHD hydroxylates HIF-1α and HIF-2α, triggering their interactions with the von Hippel Lindau (VHL) E3 ubiquitin ligase complex marking the proteins for proteasomal degradation. In contrast, stabilization of HIF-1α and HIF-2α in hypoxia leads to expression of genes involved in compensating physiological pathways such as angiogenesis, glucose utilization, cell proliferation, and tumor progression (67). We hypothesized that FQ inhibition of PHD by iron chelation would therefore stabilize HIF-1α and HIF-2α. Control experiments involved cells treated with known iron antagonists DFO (a siderophore) or cobalt chloride (CoCl_2_), a divalent metal ion competitor for dioxygenase binding. In both cases, HIF-1α and HIF-2α levels increased as expected (Fig. 9). It was therefore completely unexpected that cells treated with CIPRO, ENRO, or NOR showed a profound decrease in HIF-1α and HIF-2α levels relative to controls (Fig. 9, *A* and *B*). Remarkably, HIF-1α and HIF-2α were suppressed in CIPRO-treated cells even upon co-treatment with DFO or CoCl_2_ in hypoxia (Fig. 9, *C–E*). These results suggest that FQs exert suppressive effects on HIF-1α and HIF-2α protein levels upstream of regulated proteolysis. We considered the following three possible explanations for HIF-1α and HIF-2α suppression by FQ: increased proteasomal/lysosomal HIF degradation by a VHL-E3 ligase-independent mechanism, inhibition of HIF gene transcription, or inhibition of HIF mRNA translation.

##### FQs Inhibit JNK1 Activity

**FIGURE 10.**
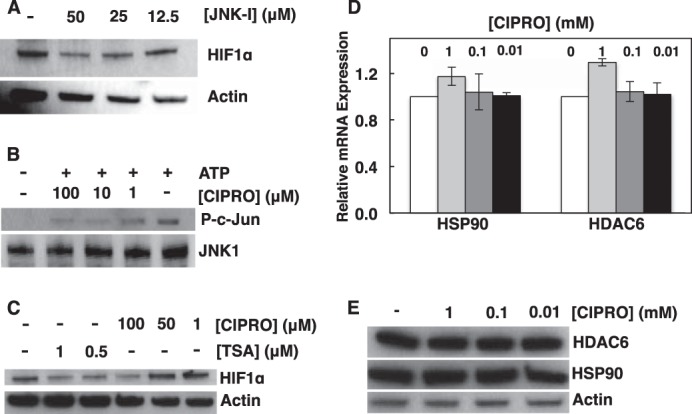
**FQs inhibit JNK1 activity.** *A,* HIF-1α status in HEK293 cells exposed to JNK inhibitor (JNK-1, SP600125). *B,* CIPRO inhibits JNK activity. *C,* comparison of HIF-1α status in cells treated with trichostatin-A (*TSA*-HDAC inhibitor) and CIPRO. *D* and *E,* HDAC6/HSP90 mRNA expression and protein levels in cells treated with CIPRO. Data are means ± S.D. representative of *n* ≥ 3 independent experiments.

It has been suggested that c-Jun N-terminal kinases (JNK) play a role in VHL-independent degradation of HIF proteins (68, 69). Gong *et al.* (68) found that some quinolone-derived drugs bound strongly to the ATP binding pocket of JNK1. Zhang *et al.* (69) suggested a PHD-VHL-independent mechanism of HIF-1α degradation involving chaperone proteins HSP90 and HSP70. The proposed mechanism of HIF-1α loss involved compromised JNK1 function leading to destabilization of histone deacetylase 6 (HDAC6) and subsequent hyperacetylation of HSP90, inhibiting the chaperone (69). As HIF-1α is a known client protein of HSP90, JNK1 inhibition could lead to HIF-1α misfolding and proteasomal degradation. To investigate this model, we inhibited JNK1 in HEK293 cells using known inhibitor SP600125 and assessed HIF-1α protein levels by Western blotting (Fig. 10*A*). As predicted, HIF-1α was lost upon JNK1 inhibition. We then tested whether FQs inhibit JNK1 function. Using a commercial JNK1 *in vitro* assay, we tested the ability of JNK1 to phosphorylate client protein c-Jun in the presence of 1–100 μm CIPRO. JNK1 inhibition was observed (Fig. 10*B*). CIPRO inhibition of JNK1 tended to support the study by Zhang *et al.* (69) as a plausible explanation for FQ suppression of HIF. However, although we confirmed that HDAC6 inhibition does lead to HIF-1α loss (Fig. 10*C*), this loss of JNK1 activity was not correlated with destabilization of HDAC6 (Fig. 10, *D* and *E*). In contrast, the parent study (69) showed loss of HDAC6 mRNA and protein upon JNK1 inhibition. Furthermore, the predicted down-regulation of HSP90 transcription upon JNK1 inhibition was not detected (Fig. 10*D*). Thus, although we found CIPRO to be a JNK1 inhibitor *in vitro*, this property did not provide a clear link to suppression of HIF levels in cell culture.

##### FQs Do Not Stimulate HIF Degradation in Proteasomal or Lysosomal Pathways

**FIGURE 11.**
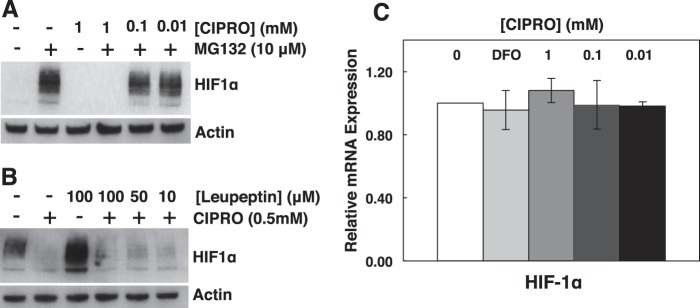
**FQ-dependent reduction of HIF-1α does not involve proteasomal or lysosomal degradation.** Inhibition of proteasomal (MG132) (*A*) or lysosomal (leupeptin) (*B*) protein degradation does not rescue HIF1α levels in cells treated with CIPRO. *C,* qRT-PCR analysis of relative HIF-1α mRNA in cells treated with DFO or CIPRO. Data are the means ± S.D. representative of *n* ≥ 3 independent experiments.

As shown in Fig. 9, FQ treatment of HEK293 cells suppresses HIF-1α accumulation independent of DFO, CoCl_2_, or hypoxia. We explored whether FQ increased HIF-1α degradation. We treated cells with CIPRO in the presence of the potent proteasome inhibitor MG132 (10 μm). The results (Fig. 11*A*) show that proteasome inhibition does not rescue HIF-1α. This finding is consistent with our findings for JNK1 inhibition. Similarly, lysosomal protease inhibition by leupeptin (100 μm) had no effect (Fig. 11*B*). Thus, HIF-1α suppression upon FQ treatment presumably involves an earlier step in HIF-1α biosynthesis.

##### FQs Inhibit HIF mRNA Translation

**FIGURE 12.**
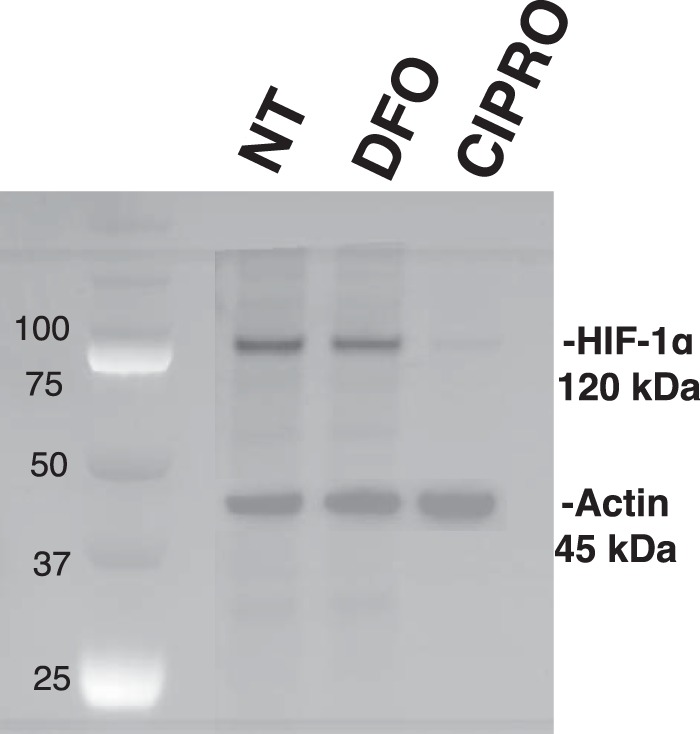
**HIF-1α mRNA translation is repressed in CIPRO-treated cells.** HIF-1α and actin immunoprecipitation after metabolic labeling with [^35^S]Met. Cell cultures were treated with 1 mm CIPRO or 100 μm DFO after methionine starvation, and then nascent proteins were radiolabeled and immunoprecipitated for further processing and imaging.

To determine whether HIF-1α gene transcription is inhibited by FQ, we quantified HIF-1α mRNA from CIPRO-treated cells by qRT-PCR. No significant change in HIF-1α transcript levels was observed (Fig. 11*C*). Combined with the prior observations that HIF-1α protein loss did not result from increased protein degradation, this result placed focus on suppression of HIF-1α mRNA translation in the presence of FQ. Evidence for such FQ-induced translation repression was observed in an experiment to measure new HIF-1α synthesis after metabolic labeling (Fig. 12). Here, we observed that FQ treatment blocked new HIF-1α mRNA translation upon addition of methionine. Actin mRNA was translated equally in both the FQ and control cells (Fig. 12).

##### FQ-induced HIF mRNA Translation Repression Is Not Linked to Microtubule Disruption

**FIGURE 13.**
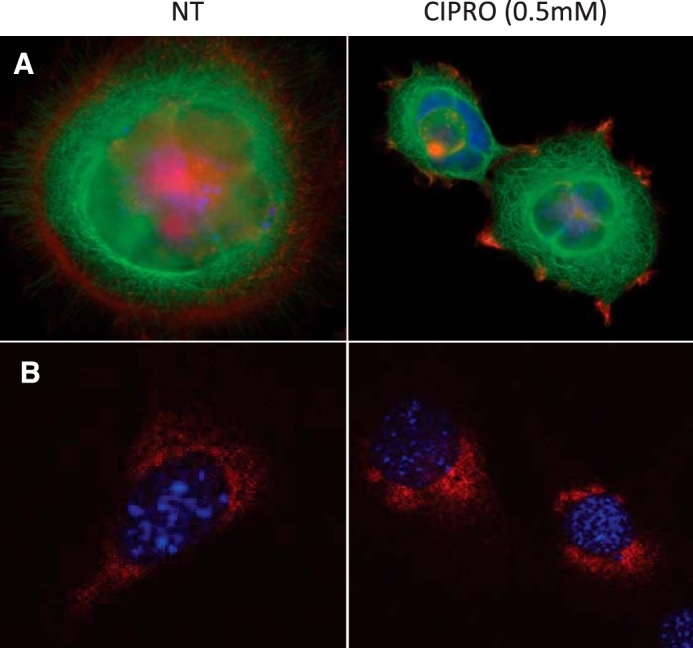
**FQs do not destabilize cell microtubules or cause HIF-1α sequestration in P-bodies.** *A,* cells stained for tubulin (*green*) and DNA (Hoechst dye; *blue*). *B,* staining for DNA (Hoechst dye; *blue*) and HIF-1α (*red* molecular beacon hybridization performed as in Ref. 44)).

Carbonaro *et al.* (70) reported HIF-1α translational repression upon microtubule disruption by certain drugs. Cells treated with taxol, a microtubule stabilizer, showed loss of HIF-1α synthesis due to HIF-1α mRNA targeting to P-bodies (70). Our analysis of microtubule structure did not show clear signs of disruption or stabilization (Fig 13*A*). Furthermore, analysis of HIF-1α mRNA distribution by immunofluorescence studies did not reveal clear aggregation patterns (Fig 13*B*), arguing against HIF-1α sequestration into P-bodies.

## Discussion

Tendinopathies represent distinctive and peculiar side effects of FQ antibiotics (71–73). This collagen pathology has been considered mysterious. Some studies have suggested extracellular matrix irregularities result from FQ enhancement of protease activities (23–25, 74, 75). Here, we suggest a different mechanism. FQs are potent iron chelators capable of inhibiting 2-KG-dependent dioxygenases because of the crucial role of iron in the active site. We show that FQ treatment inhibits collagen maturation. Prolyl 4-hydroxylase and lysyl hydroxylase are iron-dependent enzymes essential for the post-translational modification of collagen. Both play central roles in collagen maturation through hydroxylation of proline and lysine residues to mediate collagen cross-linking. Covalent cross-links are required for the tensile strength of collagen fibers (64). We suggest that it is iron chelation by FQ that accounts for suppressed collagen hydroxylation, giving rise to tendinopathies. FQs are able to chelate multiple divalent and trivalent metals (37), but the demonstration that epigenetic effects are at least partially reversible by exogenous iron suggests that iron chelation is a primary mechanism of inhibition. Moreover, transcript analysis of P4HA1 and LH1 shows clear repression upon FQ treatment suggesting additional mechanisms involved in collagen weakening. Invoking studies by Aro *et al.* (66), our surprising finding of FQ-mediated suppression of HIF-1α explains the decrease in P4HA1 and LH1 mRNA levels upon FQ treatment. Additionally, suppression of HIF-1α can have drastic effects on vascularization and energy metabolism in connective tissues, contributing to decreased blood flow in an already hypoxic and avascular tissue. We suggest that these three insults, inhibition of prolyl and lysyl dioxygenases, reduction of P4HA1 and LH1 mRNA levels, and reduced tendon vascularization upon HIF-1α depletion, together account for FQ-induced tendinopathies.

We further show that both JMHD and TET dioxygenases are inhibited by FQ treatment in cultured cells, causing histone and DNA hypermethylation. This conclusion is further confirmed by the observation that supplemental iron prevents histone and DNA hypermethylation by FQ treatment. This is the first study to show global epigenetic changes induced by FQ antibiotics. Together with FQ effects on collagen maturation, these epigenetic changes may contribute to nephrotoxicities observed in patients treated by FQ. Future studies will be needed to determine gene expression changes resulting from FQ treatment.

Given their ability to inhibit dioxygenases, we report the unexpected and counterintuitive suppression of HIFα proteins by FQ. Although FQ inhibition of PHD enzymes marking HIF-1α and HIF-2α for destruction should stabilize these proteins, both HIF-1α and HIF-2α levels were dramatically decreased upon FQ treatment. Hypoxia, DFO, or CoCl_2_ co-treatment was unable to overcome this suppression. We show that neither enhanced protein degradation nor decreased mRNA levels account for HIF-1α and HIF-2α suppression. Instead, we find that HIF mRNA translation is inhibited by FQ treatment. Future experiments will be necessary to explore pathways associated with translational suppression of HIF-1α synthesis.

To what extent are the present results relevant to therapeutic FQ doses? We observed that 2-KG-dependent dioxygenases were inhibited by FQ concentrations between 10 μm and 1 mm. CIPRO concentrations as low as 10 μm inhibited HIF mRNA translation. A CIPRO concentration of 100 μm strongly inhibited both JMHD and TET1 dioxygenases, with effects even greater at 1 mm. Comparing these concentrations to physiological concentrations reported during FQ therapy, serum concentrations of CIPRO are ∼16 μm but can approach 1–3 mm in the kidneys of treated patients (76). Upon overdose, CIPRO concentrations in the urine are even higher (29). Our choice of human embryonic kidney cells to study FQ effects reflects the high kidney exposure upon FQ treatment. Indeed, acute renal failure is associated with high FQ concentration (31). A correlation has also been observed between FQ-induced tendinopathies and chronic renal failure (77–79). Thus, we propose that iron chelation by FQ antibiotics explains tendinopathy and nephrotoxicity in part through inhibition of iron-dependent dioxygenase enzymes.

## Author Contributions

S. B. and Y. F. H. conceived and executed experiments and wrote the manuscript. L. J. M. conceived and supervised experiments and wrote the manuscript. All authors analyzed results and approved the final version of the manuscript.

## Footnotes

3 The abbreviations used are:

FQ: fluoroquinolone
BisTris: 2-[bis(2-hydroxyethyl)amino]-2-(hydroxymethyl)propane-1,3-diol
DFO: deferoxamine mesylate
CIPRO: ciprofloxacin
NOR: norfloxacin
ENRO: enrofloxacin
PHD: prolyl hydroxylase dioxygenase
JMHD: Jumonji domain histone demethylase
DFO: deferoxamine mesylate
CAS: chrome azurol S
qRT: quantitative RT
HIF: hypoxia-inducible transcription factor
NT: not treated
2-KG: 2-ketoglutarate
P4H: prolyl 4-hydroxylase
VHL: von Hippel Lindau
TET: Ten-eleven translocation
COL: collagen.
